# Subjective feeling of re‐experiencing past events using immersive virtual reality prevents a loss of episodic memory

**DOI:** 10.1002/brb3.1571

**Published:** 2020-04-27

**Authors:** Lucie Bréchet, Sebastien B. Hausmann, Robin Mange, Bruno Herbelin, Olaf Blanke, Andrea Serino

**Affiliations:** ^1^ Laboratory of Cognitive Neuroscience Brain Mind Institute School of Life Sciences Swiss Federal Institute of Technology (EPFL) Geneva Switzerland; ^2^ Center for Neuroprosthetics Swiss Federal Institute of Technology (EPFL) Geneva Switzerland; ^3^ Department of Neurology Geneva University Hospital Geneva Switzerland; ^4^ MySpace Lab Department of Clinical Neurosciences University Hospital of Lausanne University of Lausanne Lausanne Switzerland

**Keywords:** bodily self‐consciousness, episodic memory, first‐person perspective, memory preservation, virtual reality

## Abstract

**Introduction:**

Personally meaningful past episodes, defined as episodic memories (EM), are subjectively re‐experienced from the natural perspective and location of one's own body, as described by bodily self‐consciousness (BSC). Neurobiological mechanisms of memory consolidation suggest how initially irrelevant episodes may be remembered, if related information makes them gain importance later in time, leading for instance, to a retroactive memory strengthening in humans.

**Methods:**

Using an immersive virtual reality system, we were able to directly manipulate the presence or absence of one's body, which seems to prevent a loss of initially irrelevant, self‐unrelated past events.

**Results and Conclusion:**

Our findings provide an evidence that personally meaningful memories of our past are not fixed, but may be strengthened by later events, and that body‐related integration is important for the successful recall of episodic memories.

## Graphical abstract

Using an immersive virtual reality system, we were able to retroactively create the subjective feeling of having a self‐related experience of “being in there,” which seems to prevent a loss of initially irrelevant, self‐unrelated past events. Our findings provide an evidence that personally meaningful memories of our past are not fixed, but may be strengthened by later events, and that body‐related information is an important mechanism for episodic memories.

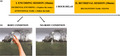

## 1 INTRODUCTION

Most events that we experience during our daily life will be forgotten (Hupbach, Hardt, Gomez, & Nadel, 2008; Talamini & Gorree, 2012). We form detailed and lasting memories only for a small part of our everyday events. What determines which events will be remembered and which lost? Episodic memories (EM) are personally meaningful and related to our sense of self (Tulving, 2002). EM comprise precise information about the place, the time as well as the content of self‐relevant, past events. We tend to remember salient past events, because the information may be relevant to us in the future, and forget whatever is less self‐relevant. However, we do not always know when an important event may happen. For example, the stranger who asked for directions becomes more relevant, and thus better remembered, after you realize that your wallet is missing. Synaptic and behavioral tagging hypotheses have suggested a neurobiological mechanism of memory consolidation, by which initially unstable and weak memories are retroactively strengthened by conceptually related, that is, category‐specific strong events (Frey & Morris, 1997; Moncada, 2007; Nomoto et al., 2016; Redondo & Morris, 2011; Wang, Redondo, & Morris, 2010). In a recent behavioral study (Dunsmoor, Murty, Davachi, & Phelps, 2015), authors showed that if participants received an electric shock while looking at a neutral picture, they remembered better those pictures seen prior to receiving the electric shock, which were conceptually related compared to those that participants have seen prior to receiving the electric shock that were conceptually unrelated. Thus, it is crucial to temporarily store apparently irrelevant events in memory (e.g., seeing a picture of an animal), in case these unimportant past events may gain unexpected importance (i.e., receiving an electric shock when seeing another animal) later in time.

Every event in our lives is experienced and thus encoded from the natural location (self‐location) and perspective (first‐person perspective, 1PP) of a body that we feel as our own (self‐identification) (Bergouignan, Nyberg, & Ehrsson, 2014; St. Jacques, Szpunar, & Schacter, 2017). Subjective re‐experiencing of personal past events is also described from the same embodied viewpoint and location as during the event's encoding. Thus, both encoding and retrieval of episodic memories are linked to “embodied” components of self‐experience that together have been defined as bodily self‐consciousness (BSC, (Blanke & Metzinger, 2009)). Several lines of work have shown that BSC depends on the continuous, prereflexive, and coherent processing of multisensory bodily signals (Blanke, 2012; Blanke & Metzinger, 2009; Blanke, Slater, & Serino, 2015). Accordingly, manipulating the coherence of multisensory bodily cues, such as during the rubber hand illusion or the full body illusion, affects the different components of BSC (Ehrsson, 2012; Serino, 2019; Serino et al., 2013; Tsakiris, Longo, & Haggard, 2010). Studies started to directly explore the link between BSC and EM and showed, for instance, that manipulating the direction of the 1PP, from an embodied versus a disembodied perspective at the encoding, affects future recall of the event (Bergouignan et al., 2014). In a recent study (Bréchet et al., 2019), we have manipulated the congruency between multisensory visuomotor bodily cues at the encoding and tested its effect on later recognition: participants explored a virtual reality (VR) environment while viewing or not viewing their body in the virtual scene. Delayed recognition of events was higher when they were encoded with a body than without a body present in VR, indicating a further link between the subjective experience of being present here and now, that is, BSC, and future re‐experiencing of the past event, that is, EM. These previous findings show a proactive effect on memory performance due to the congruency of bodily signals. In the present study, we tested whether the presence versus absence of one's body at the encoding could also affect retroactively memory for conceptually related events.

To this aim, we adapted a paradigm recently used by two behavioral studies, demonstrating how memory for neutral images of a specific category (i.e., animals or tools) can be strengthened by future fearful (Dunsmoor et al., 2015) or rewarding (Patil, Murty, Dunsmoor, Phelps, & Davachi, 2017) events. In those studies, during the preconditioning classification phase, participants were presented with two neutral categories of images depicting animals and tools that appeared to be of the same relevance. However, during the second phase of the incidental encoding, the so‐called “conditioning classification,” a salient emotional event, either fear conditioning (electric shock (Dunsmoor et al., 2015)) or reward motivation (earning money (Patil et al., 2017)), became associated with only one of the two categories of images. As a result, during a later memory recognition task, participants remembered better not only objects that were associated with fear or reward during the conditioning phase, but surprisingly also the conceptually related images from the preconditioning phase. These results suggest that meaningful events can selectively consolidate memory for prior, seemingly insignificant information at the time of encoding.

Here, we tested whether presence versus absence of one's own body, which we created using immersive VR at the second phase, could act as a “conditioning classification” trigger affecting recognition for conceptually related scenes from the first phase of exposure. The main motivation of our study is to show that relating neutral stimuli to the bodily self (rather than electric shocks or reward) may retroactively enhance episodic memory. A substantial amount of evidence previously suggested that memory is enhanced when people associate neutral stimuli to themselves rather than others, the so‐called “integrative self model” (Sui & Gu, 2017; Sui & Humphreys, 2015). Here, we go a step further and suggest that the integration of multisensory bodily signals, particularly the congruency between multisensory visuomotor bodily cues at the encoding, may enhance episodic memory (Park & Blanke, 2019).

We used a novel form of mixed VR that allowed us to “immerse” participants in real‐life‐like scenarios, while being able to control each element in the scene, including the presence of participants’ physical bodies. We hypothesized, in line with our previous finding, that the presence of one's body would result in a better performance in the recognition task for items previously presented in the conditioning phase (experiment 1). More importantly, in line with the synaptic and behavioral tag hypothesis, we also predicted a better recognition for items conceptually related to the preconditioning phase (experiment 2), that is, items belonging to same category that were shown with the body present during the conditioning phase, while no improvement was expected for items from the category in which the body was not present. We report results from two experiments. In the first experiment, we replicate our recent study (Bréchet et al., 2019) and confirm that the presence of one's own body in the VR environment influenced the accuracy in remembering what participants have and have not encoded. The main goal of our study is the second experiment, which uses a novel retroactive paradigm and combines our previously created inside rooms (tested in Bréchet et al., 2019) and outside scenes (tested in experiment 1). In the second experiment, we demonstrate the effectiveness of the presence of the body in the scene and suggest its role as a behavioral tag affecting retroactively episodic memory.

## 2 EXPERIMENT 1

### 2.1 Methods

#### 2.1.1 Participants

The sample of experiment 1 consisted of 15 right‐handed participants (*M* = 26.1, *SD* = 3.6, 10 female). We excluded one participant because of chance‐level memory performance. All participants had normal or corrected‐to‐normal vision and had no current diagnoses or history of psychological or neurological disorders. Informed consents were obtained from all our participants. The study was approved by the local ethical committee, and the two experiments were conducted in conformity with the Declaration of Helsinki.

#### 2.1.2 Reality Substitution technology (RealiSM)

We created realistic outside scenes, from which participants could see their own hands, trunk, and legs from 1PP. Participants felt immersed into the prerecorded scenes and seeing themselves present there. The VR technology included spherical capture and recording system for 1PP simulations of real‐life environments. Sixteen cameras and microphones covered the whole sphere of perception around a viewpoint (over 360° horizontally and vertically, stereoscopic vision, binaural panoramic audio). RealiSM software combined all data into a high‐resolution panoramic audiovisual computer format (equivalent to more than four stereoscopic full HD movies). A head‐mounted display (HMD, 640 × 800 resolution, 110° diagonal field of view; Oculus Rift Development kit; Oculus VR) was used to immerse participants into the prerecorded outside scenes. Stereoscopic depth cameras were attached on the HMD to capture participants’ bodies (i.e., cameras were on/off) from the 1PP. The VR technology was used both in experiments 1 and 2.

#### 2.1.3 Procedure

**Figure 1:**
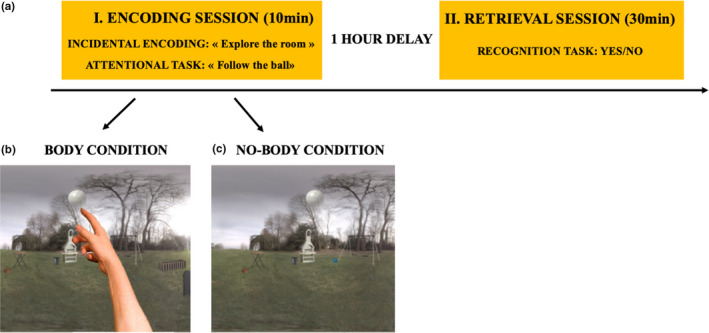
(a) Paradigm of experiment 1. First, participants incidentally learned the context of two different outside scenes (i.e., encoding session; 10 min). Participants were immersed back into the scenes with one‐hour delay and were asked to perform a recognition task and to rate their subjective confidence in remembering each scene (i.e., recognition session; 30 min). (b) Body condition. Physical bodily self‐manipulation in the prerecorded outside scene. Participants were asked to point with their finger at the moving ball. Participants experienced the feeling of being physically present in the outside scene as they had the visual feedback of seeing their body. (c) Nobody condition. Participants were physically pointing at the ball, but there was no visual feedback of their body

Here, we expanded the experimental setting of inside rooms from our prior study (Bréchet et al., 2019) to outside scenes into which participants were immersed during two stages (encoding and recognition) of EM testing. We hypothesized that the memory for contextual details of the outside scenes in one‐hour delay memory recognition task would increase in the body‐present compared to body‐absent condition. The experiment consisted of two sessions, an incidental encoding task followed by one‐hour delayed surprise memory recognition task. The procedure is depicted in Figure 1a.

##### Encoding session

Participants were immersed into two outside scenes that were prerecorded and played via HMD. To familiarize with the VR technology, participants were first immersed into a scene for 5 min. We specifically asked the participants to remain seated, turn and look around, and explore the environment. After the VR familiarization, a ball appeared in each of the two outside scenes and started to freely move around for 30 s. Participants were asked to visually follow a movement of the ball. This attention task was created in order to assure that participants fully explored both of the 360° prerecorded scenes. Moreover, participants were asked to follow the trajectory of the ball by physically pointing at the moving ball with their finger. The trajectory was carefully selected, so it would not directly cover the view of the objects in the prerecorded scenes, but that the VR ball would pass close to all the objects in order to assure that participants would not miss seeing one. Ten daily‐life objects were positioned in each scene during the memory encoding to create the most natural, everyday‐like environment as possible. For example, there was an outside grill and a bench in the park, children bikes nearby. Each scene included a different set of objects in order to keep the same level of novelty and to avoid any facilitation on the following recognition task. The main manipulation (i.e., the presence or absence of one's physical body) was accomplished with the use of the stereoscopic depth cameras (i.e., turning them on/off), mounted on the HMD, to capture in real‐time participants’ bodies from the 1PP. In one scene, participants could see their physical hand, trunk, and legs; hence, there was visual feedback of their physical body (Figure 1b). In the other scene, there was no visual feedback of participant's body in the scene (Figure 1c). The order of presentation of the body and nobody condition was counterbalanced between participants. Encoding was incidental; therefore, participants were not informed that they were performing a memory task. Instead, participants thought the experiment was about exploring new virtual reality settings, which allowed us to test how much we remember of everyday‐life events compared to, for example, asking participants to memorize a list of words.

##### Recognition session

One hour after the encoding session, participants revisited the same outside scenes they explored during the encoding. There were three blocks of 40 trials, each trial lasting 10 s. Within the three blocks of 40 trials, 10 trials were presented as exactly the same as during the original encoding session (i.e., including the same previously presented everyday‐life 10 objects). Thirty trials were modified and presented with either 1, 2, or 3 new objects replaced by the old ones. The position of both old and new objects remained always the same. The blocks and individual trials were presented in a randomized order. Participants were free to re‐explore each outside scene for 10 s, after which they were asked whether each scene looked exactly the same as when they first saw it. The instructions were displayed on a black background of the HDM display. Participants were holding a wireless computer mouse, by which they provided the answers (i.e., yes/no).

#### 2.1.4 Statistical analysis

We tested whether the presence or absence of one's own body would affect participants’ accuracy to distinguish between the old versus new objects. Furthermore, to test participants’ ability to discriminate between the number of objects changed (i.e., 1 object, 2 objects, or 3 objects), 2 × 3 repeated measures ANOVAs on the sensitivity measure (*d*′) and response bias (*c*) were performed (Stanislaw & Todorov, 1999). Where appropriate, Greenhouse–Geisser corrections of degrees of freedom were used in the statistical analyses. Significant ANOVA effects were explored by post hoc tests using Bonferroni correction. The significant level in all statistical tests was set to alpha 0.05. All means are reported alongside a 95% confidence interval (CI), and all significant test statistics are accompanied by partial *η* ^2^ or Cohen's *d* effect sizes.

#### 2.1.5 Results

**Figure 2:**
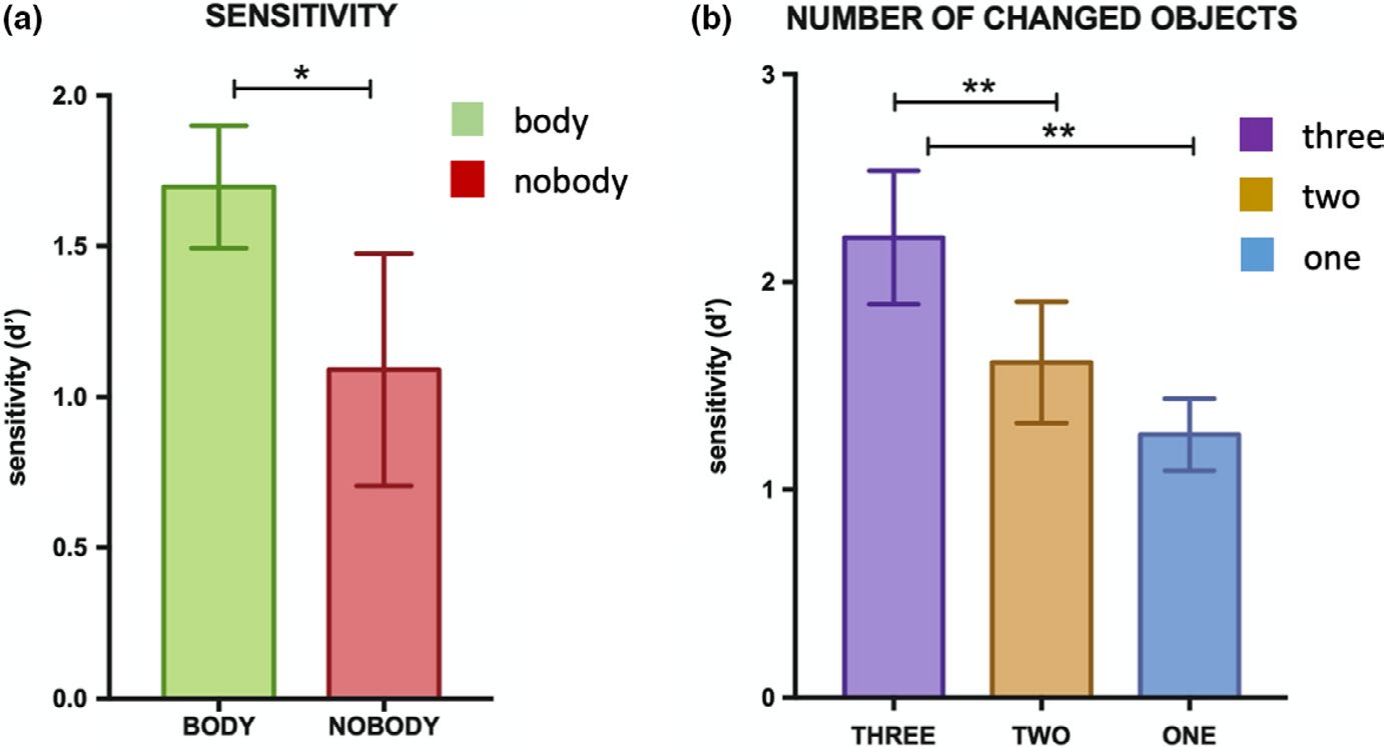
Signal detection measures in experiment 1 (body vs. nobody, one‐hour delay memory recognition task). (a) Mean sensitivity (*d*′) measure for body versus nobody condition; (b) Mean sensitivity (*d*′) measure for the number of changed objects. EM performance is indicated in *d*′ + *SEM*. (**) *p* < .01; (*) *p* < .05

Participants were significantly more accurate (*d*′) in detecting changed items in the body condition (*M* = 1.69, 95% CI = [1.18, 2.2]) than in the nobody condition (*M* = 1.08, 95% CI = [0.28, 1.84]); *F* (1, 13) = 4.96, *p* = .04, partial *η* ^2^ = 0.27 (Figure 2a). Further, the sensitivity analysis to distinguish the number of objects changed (i.e., 1 object, 2 objects, or 3 objects) revealed a statistically significant main effect; *F* (2, 26) = 12.67, *p* = .0001, partial *η* ^2^ = 0.49. Pairwise comparisons revealed that participants could accurately distinguish between 1 and 3 objects changed (*p* = .003, Bonferroni corrected) with a mean difference of 0.74, 95% CI = [0.26, 1.22]. Participants were also sensitive in discriminating between 2 and 3 objects (*p* = .005, Bonferroni corrected) changed with a mean difference of 0.43, 95% CI = [0.74, 1.31] (Figure 2b). The other analyses including the interactions and response bias were not significant. In the first experiment, we demonstrate that the presence of congruent multisensory cues from one's body enhances the EM accuracy in remembering everyday‐life items not only in inside rooms, as we have shown recently (Bréchet et al., 2019), but also in outside scenes.

## 3 EXPERIMENT 2

### 3.1 Methods

#### 3.1.1 Participants

The study sample of experiment 2 consisted of 16 right‐handed participants (*M* = 25.3, *SD* = 6.2, 10 female). Inclusion criteria were the same as for experiment 1.

#### 3.1.2 Procedure

**Figure 3:**
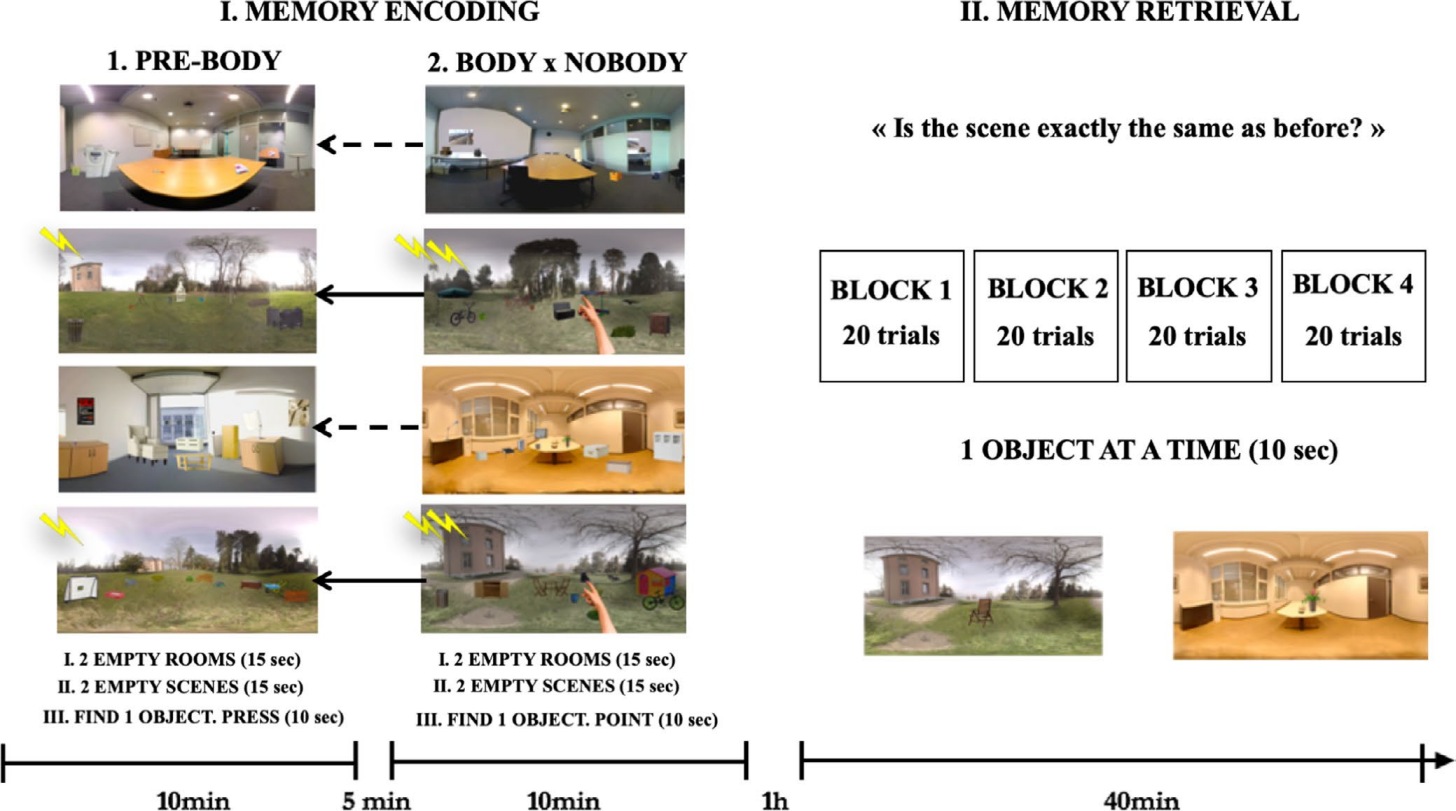
Paradigm of experiment 2. First, participants explored two empty rooms and two empty scenes without any object for 15 s each. Participants were then instructed to find daily‐life objects (40 total) belonging to inside rooms (20 objects) and outside scenes (20 objects) and press a button once they found it during the prebody conditioning (phase 1). During the body/nobody condition (phase 2), participants explored two new empty rooms and two new empty scenes without any object for 15 s each. Participants were then instructed to find new set of daily‐life objects (40 total) belonging to inside rooms (20 objects) and outside scenes (20 objects) and point toward the object once they found it. During the memory recognition, participants were asked to recognize each object as new or old

Here, we tested whether the presence of the body in VR represents a self‐relevant, strong cue that would retroactively enable the consolidation of previously encoded memories and thus prevent their loss. The experiment started with an incidental encoding task, which consisted of two phases: the prebody versus prenobody phase (phase 1) and the body versus nobody phase (phase 2). The encoding session followed by one‐hour delayed surprise memory recognition task. The procedure is depicted in Figure 3.

##### Encoding session

Participants were asked to explore 2 empty inside rooms and 2 empty outside scenes for 15 s each. Participants were then immersed into the same rooms and outdoor scenes again. There was one object at the time appearing in each environment for 10 s. Participants were asked to find the object and press a button of a wireless computer mouse if they found it. In total, there were 40 objects (10 per each room/scene) presented during the prebody phase (phase 1). After 5 min break, participants were immersed into two novel empty inside rooms and 2 novel empty outside scenes for 15 s. During this body versus nobody phase (phase 2), participants were first asked to explore the four new empty environments. After the 15 s, participants were immersed into the same rooms and outdoor scenes again. There was one object at the time appearing in each environment for 10 s. In total, there were 10 objects per room/scene. Participants were asked to find the object and physically point at it if they found it. The main manipulation (i.e., the presence or absence of one's physical body) was specific to either two inside rooms or two outside scenes in phase 2. Thus, either both inside rooms were presented with physical bodies present, while both outside scenes did not include the physical bodies or vice versa. Crucially, in the body condition, participants could see their physical hand, trunk, and legs while physically pointing at the objects in either the rooms or scenes, while in the nobody condition only the scene, without the body was visible. The presentation of the body versus nobody condition was counterbalanced between participants.

##### Recognition session

After one‐hour delay, participants were informed that they would revisit the same outside scenes and inside rooms again in four blocks of 20 trials, each trial lasting 10 s. The blocks and trials were randomized. Within the four blocks of 20 trials, 10 trials were presented with exactly the same 10 objects (old) as during the original encoding session and 10 trials with 10 new objects. Participants were free to re‐explore each outside scene and inside room for 10 s, after which they were asked a question. The instructions were displayed on a black background of the HDM display. Participants were asked whether the scene was exactly the same as before. Participants were holding a wireless computer mouse by which they provided the answers (i.e., yes/no).

#### 3.1.3 Statistical analysis

In order to test the effects of the body condition (i.e., body present vs. body absent) and the phase (prebody condition vs. body x nobody condition) on the memory for contextual details of real‐life‐like events, repeated measures 2‐way ANOVAs on the sensitivity measure (*d*′) and response bias (*c*) were performed (Stanislaw & Todorov, 1999). Follow‐up *t* tests on the sensitivity measures (*d*′) were conducted separately for each phase (phase 1 and phase 2). Where appropriate, Greenhouse–Geisser corrections of degrees of freedom were used in the statistical analyses. Significant ANOVA effects were explored by post hoc tests using Bonferroni correction. The significant level in all statistical tests was set to alpha 0.05. All means are reported alongside standard error of a mean, and all significant test statistics are accompanied by partial *η* ^2^ or Cohen's *d* effect sizes.

#### 3.1.4 Results

**Figure 4:**
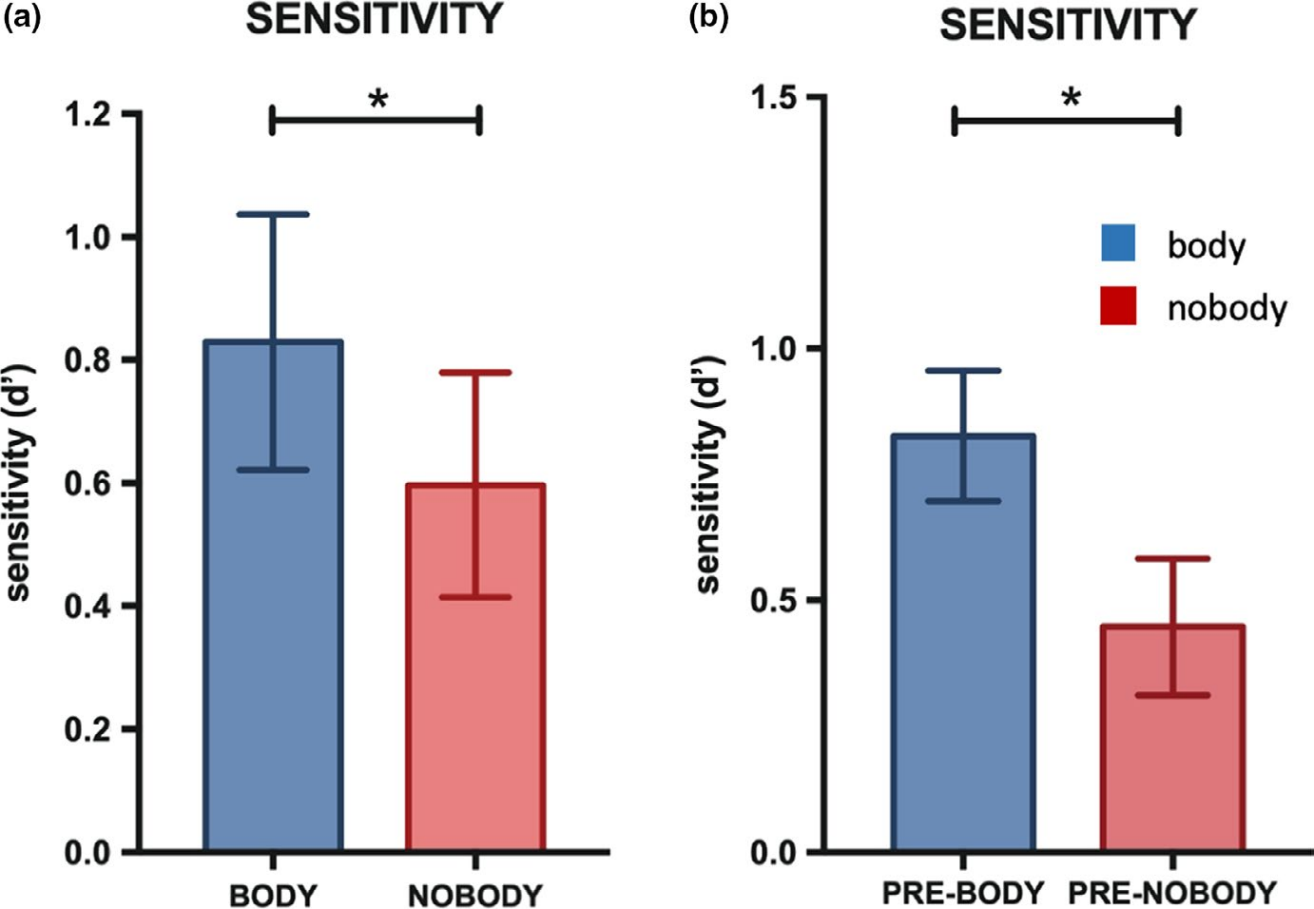
Signal detection measures in experiment 2 (one‐hour delay memory recognition). (a) Body versus nobody mean sensitivity (*d*′) (phase 2). (b) Preconditioning/prebody mean sensitivity (*d*′) (phase 1); EM performance is indicated in *d*′ + *SEM*. (**) *p* < .01; (*) *p* < .05

Two‐way ANOVA with body (i.e., body and nobody) and phase (i.e., prebody and prenobody) as factors revealed a significant main effect of body condition (*F* (1, 14) = 9.91, *p* = .007, partial *η* ^2^ = 0.41) using the sensitivity measure (*d*′). The other main effect and interactions, including the response bias, were not significant. Post hoc *t* test revealed, as predicted from the results of experiment 1 and our previous study (Bréchet et al., 2019), that the sensitivity measure was higher in the body condition (*M* = 0.99, 95% CI = [0.71, 1.27]) versus the nobody condition (*M* = 0.74, 95% CI = [0.51, 0.98]) for items shown in phase 2 (*t* (14) = 2.56, *p* = .02, *d* = 0.55) (Figure 4a). More importantly, the body effect on memory extended retroactively and selectively to the conceptually related objects that were observed in the prebody condition (phase 1), even if the body was not shown at that phase. Specifically, the post hoc *t* test revealed that sensitivity measure was higher in the prebody (*M* = 0.82, 95% CI = [0.54, 1.1]) as compared to the prenobody condition (*M* = 0.44, 95% CI = [0.15, 0.73]); (*t* (14) = 2.78, *p* = .01, *d* = 0.73 (Figure 4b), even if at that phase of encoding, there was no difference between the two categories of stimuli.

## 4 DISCUSSION

There is an increasing interest in the relationship between how we experience ourselves in the environment “here and now,” that is, BSC (Blanke, 2012; Blanke et al., 2015), and how we re‐experience past events in time, that is, the autonoetic component characterizing EM (Tulving, 1993). Here, we report novel findings showing that memory for seemingly insignificant everyday details of neutral real‐life‐like events can be retroactively consolidated and preserved, if future conceptually related details acquire salience by the presence of one's own physical body. Recent behavioral studies (Dunsmoor et al., 2015; Patil et al., 2017) showed that emotional learning (aversive as well as appetitive) can retroactively and selectively strengthen memories for conceptually related neutral events. However, differently from those previous laboratory‐based episodic memory studies, here we used a novel VR technology, which enabled us a) to immerse our participants and their physical bodies into complex real life inside rooms and outside scenes and b) to experimentally control both stages of memory encoding and recognition so to manipulate the presence or absence of participants’ own bodies in the VR scenarios. The present results suggest that the memory for real‐life‐like events is affected by self‐related bodily cues, as we show that the memory for episodic events is better if those are encoded while one's body is present in the scene as compared to when the body is absent. Most strikingly, here we indicate that encoding new scenes while experiencing one's own body retrospectively enhanced later memory for scenes of the same category, that were previously encoded without the body. Such effect did not occur for repeated encodings without the body, that is, for outside scenes or inside rooms that were not “tagged” by the body condition. Recently, a mechanistic function of self has been proposed, where self‐reference enhances the binding of information, such as visual features in perception (Sui & Humphreys, 2015). For example, a classification of self faces is faster than faces of strangers due to an enhanced self‐referential processing and integration (Ma & Han, 2010). Supporting the idea of an integrative self, it has been further suggested how the self‐experience of being an agent of one's own actions (Gallese & Sinigaglia, 2010) can act as a binding mechanism of the self‐related processing. Besides the rare illusions which can occur in healthy and pathological population, we experience ourselves as being located within our own body (James, 1890). The presence of one's own body thus represents “the default mode for self‐experience.” We thus speculate that the body condition at the encoding reflects the normal processing that facilitates memory consolidation. On the other hand, the nobody condition represents a level of conflict that results in limited memory consolidation. In this work, we did not study whether somebody else's body compared to one's own body would also influence the memory performance or not; however, in future studies, we aim to extend these types of findings and specifically address the self versus other body ownership factor. Additional research is needed to better understand the extent to which body ownership may influence episodic memory. Using this novel VR methodology could prove useful in the episodic memory retention, especially in elderly population.

Previous lines of research succeeded in experimentally manipulating multisensory bodily stimuli to induce changes in distinct components of BSC, including self‐identification, self‐location, and 1PP (Blanke, 2012; Blanke et al., 2015). For instance, tactile stimulation of a participant's hand or body coupled with spatially and temporally synchronous stroking of a viewed virtual hand or body gives rise to illusory self‐identification and illusory self‐location over a virtual body (Ehrsson, Holmes, & Passingham, 2005; Lenggenhager, Tadi, Metzinger, & Blanke, 2007; Petkova & Ehrsson, 2008). The present findings suggest that manipulating multisensory bodily cues—in this case, the congruency between movement, proprioceptive, and visual bodily cues in VR at the encoding—affects memory recognition, that can be seen as BSC in time (Buckner & Carroll, 2007; Liberman & Trope, 2008; Schacter, Addis, & Buckner, 2007). Thus, low‐level multisensory and motor self‐related cues affect what is normally considered higher‐level cognitive processes related to the self, such as EM (Bréchet et al., 2019).

Results from experiment 2 make a step further by showing that bodily cues retroactively preserve the loss of memory for previously encoded episodic events, suggesting a link between multisensory bodily views and memory consolidation. It seems to be relevant to temporarily store inconsequential details of events, in case these details may gain saliency later in time. Previous work (Frey & Morris, 1997) suggests this is the case because initially weak and unstable memories are tagged for later stabilization by long‐term potentiation (LTP) processes. This neurobiological mechanism has been extended to show in rats how weak episodes of training, which would be forgotten, may be stored in long‐term memory if then associated to a novel, salient experience, the so‐called synaptic‐behavioral tagging (Ballarini, Moncada, Martinez, Alen, & Viola, 2009; Moncada, Ballarini, & Viola, 2015; Wang et al., 2010). Only recently the behavioral tagging effect has been shown in humans (Dunsmoor et al., 2015; Patil et al., 2017), by demonstrating that previous events, that were conceptually related to following emotionally salient experiences, became selectively enhanced, while other previous unrelated information did not benefit from the retroactive strengthening, despite they were encoded at the same time. This effect was found only with delay, but not immediately after the encoding (similarly to humans Dunsmoor et al., 2015), suggesting the importance of postencoding consolidation processes. The hippocampal consolidation processes usually start at approximately one hour (Dudai, Karni, & Born, 2015; McGaugh, 2015; Moscovitch, Cabeza, Winocur, & Nadel, 2016; Squire, Genzel, Wixted, & Morris, 2015). Thus, we argue that the enhanced self‐relevance and recruitment of BSC‐related processing improved the consolidation process of long‐term episodic memories only with this time‐delay. Similarly, previous evidence (Sharot, Martorella, Delgado, & Phelps, 2007) showed that emotion had no effect on immediate memory recall, but only delayed memory retrieval. It would be of an interest, similarly to previous studies (Dunsmoor et al., 2015; Patil et al., 2017) to test different delay times (6 and 24 hr) in the future studies.

While the integrative self model (Sui & Gu, 2017; Sui & Humphreys, 2015) suggests how self‐reference affects information processing and the binding of memory to source (for example, how neutral stimuli evaluated in relation to self may enhance associated cognitive processes), our recently proposed BSC mechanism (Park & Blanke, 2019), which is underlying self‐consciousness, integrates specific interoceptive and exteroceptive multisensory bodily signals that relate to particular aspects of BSC, such as self‐identification or self‐location. Both models are compatible and complimentary in proposals how self‐related and cognitive networks interact and influence each other. Based on these two models, which suggest that self‐representations are fundamental to modulations of cognitive processes, we here argue that one's own body, instead of electric shock or reward, can also act as a behavioral tag for memory consolidation. More specifically, we speculate that enhanced self‐relevance and recruitment of BSC‐related processing improves not only the memory for contextual details of real‐life‐like events, but also the contextual details of seemingly irrelevant events at the time of encoding, which gained importance only later in time, when tagged by the presence of one's own body in the scene.

Finally, the present results might also have interesting implications for VR technologies. In real‐life contexts, the issue of having versus not having a body never occurs, since we experience “the same old body, always there” (James, 1890). VR technologies allow us manipulating the amount and the coherence of bodily related cues; however, most common VR setups, using HMDs, do not provide any visual cues related to the user's body in the scene. In this study, we critically used the VR technology to directly manipulate the presence or absence of one's own body, thus we speculate that we were able to modulate the feeling of being present in the virtual scene. However, the subjective feeling of presence was not measured directly and thus it remains currently unknown to what extent it is the factor that influences episodic memory. Future work is needed to measure directly the subjective feeling of presence together with larger sample size in order to increase the significance level of the findings. We acknowledge that the small sample size is an important limitation of this study and future work replicating our results with larger sample sizes is needed. The present results show that this bodily manipulation in the artificially created VR environment induces significant memory effects. These findings should be taken into account for future applications of VR technologies for entertainment (Anguera et al., 2013) or healthcare (Kyaw et al., 2019).

## CONFLICT OF INTEREST

None declared.

## AUTHOR CONTRIBUTION

L.B., R.M., and A.S. designed the experiments; L.B., S.H., and R.M. conducted the experiments; L.B., S.H., and A.S. analyzed the data; L.B. and A.S. wrote the paper. B.H. supervised the development of RealiSM technology. O.B. supervised the project.

## Data Availability

Requests for the data or materials can be sent via email to the lead author at olaf.blanke@epfl.ch.
